# Circadian rhythm lncRNA *NRON* is dysregulated in autism spectrum disorder: an observational study

**DOI:** 10.1016/j.bbrep.2026.102698

**Published:** 2026-07-02

**Authors:** Asal Ziaei, Zeinab Shirvani-Farsani, Soudeh Ghafouri-Fard

**Affiliations:** a Department of Cell and Molecular Biology, Faculty of Life Sciences and Biotechnology, Shahid Beheshti University, Tehran, Iran; b Department of Medical Genetics, School of Medicine, Shahid Beheshti University of Medical Sciences, Tehran, Iran

**Keywords:** *NRON*, *HULC*, *LINC01138*, Long non-coding RNA, Circadian rhythms, Dopamine signaling pathway, Autism spectrum disorder

## Abstract

Autism spectrum disorder (ASD) is a complex neurodevelopmental disorder. It is marked by difficulties in social communication and the presence of repetitive behaviors. Research has found that disruptions in the body's circadian rhythm and dopamine signaling pathways can contribute to the development of this disorder. Long non-coding RNAs (lncRNAs) are important for regulating gene expression. They have been identified as potential risk genes and diagnostic markers for ASD. This observational (case-control) study aimed to investigate and compare the expression levels of three lncRNAs related to circadian rhythm, *NRON*, *HULC*, and LINC01138, and two lncRNAs linked to dopamine signaling, *MIAT* and *TRAF3IP2-AS1*, in the peripheral blood of 30 patients with ASD and 41 healthy controls using Real-time PCR. The results showed a significant increase in *NRON* lncRNA expression in patients with ASD compared to healthy controls (P = 0.0048**). However, the expression level of *HULC*, LINC01138, *MIAT*, and *TRAF3IP2-AS1* did not show any significant difference between these two groups (P > 0.05). Additionally, when analyzing by sex, affected males had significantly higher *NRON* expression than control males (P = 0.002**). There was also a significant correlation between the expression of *MIAT* and *TRAF3IP2-AS1* and the age of ASD patients. Moreover, ROC curve analysis suggested that *NRON* could serve as a diagnostic marker for ASD (AUC = 0.70 and P = 0.004). Overall, these findings indicate that dysregulation of *NRON* in peripheral blood is associated with ASD. While the functional relevance of this peripheral signature to CNS pathophysiology remains to be established, *NRON* may serve as a candidate for future peripheral biomarker discovery attempts.

## Highlights

• We found a significant increase in *NRON* lncRNA in ASD patients.
• Affected males had significantly higher *NRON* expression than control males.
• Expressions of *MIAT* and *TRAF3IP2-AS1* were correlated with the age of patients.
• *NRON* could serve as a diagnostic marker for ASD.
• Dysregulation of *NRON* may be involved in the development of ASD.

## 1 Introduction

Autism spectrum disorder (ASD) is a neurodevelopmental condition that is known as a spectrum disorder which includes a variety of symptoms with different severities. ASD is a heterogeneous disorder in which multiple genetic and non-genetic factors, individually or in combination, contribute to its development [[1], [2], [3]].

The roles of circadian rhythm disturbances and dysregulation of dopamine signaling pathways in the etiopathogenesis of ASD have been subjects of interest and investigation in recent years. Circadian rhythms in mammals are 24-h rhythms that are observed in a wide range of biological processes and are maintained by circadian or biological clocks [4]. The central clock in the suprachiasmatic nucleus (SCN), in turn, partially entrains the peripheral circadian clock via neural and endocrine pathways and coordinate peripheral clocks with the day/night cycle. Genetic and epigenetic changes in the main elements of the circadian pathway indicate the pivotal role of circadian disruption in the pathogenesis of ASD. However, the exact mechanism by which circadian pathways influence the risk and progression of ASD remains to be fully understood [5]. Dopamine (DA), as a major monoamine neurotransmitter in the brain, has a key role in higher brain functions, and dysfunction of dopaminergic signaling has been implicated in various psychiatric disorders. The DA system is involved in ASD; and disruption of dopaminergic transmission in the midbrain is the root of traits such as social deficits and stereotyped behaviors in ASD [6].

Several non-coding RNAs have indicated functional association with circadian rhythms and Dopamine [7,8]. Long non-coding RNAs (lncRNAs) are a group of non-coding transcripts with a length of more than 200 nt. lncRNAs are regulatory ncRNAs that act as decoys, signals, guides, scaffolds, etc and spatial and temporal expression patterns of these transcripts have underlined their diverse regulatory functions including regulating gene expression, and shaping the chromatin architecture in neurological disorders [[9], [10], [11]]. On the other hand, lncRNAs can be used as predictive and diagnostic biomarkers for diseases related to the nervous system such as ASD, which enable the objective identification of this disorder and can provide more effective treatment strategies [12,13]. The exploration of neuropsychiatric disorders has been transformed by multi-omics technologies. Genomics, transcriptomics, and proteomics have collectively identified numerous candidate genes and pathways implicated in ASD [14]. In this complex outlook, identifying central “hub” genes or molecules that lie at the convergence of multiple disrupted pathways is a key goal, as they represent high-priority targets for diagnostic and therapeutic development [15]. Our study focuses on a select group of lncRNAs that lie at the intersection of circadian rhythm and dopamine signaling, two pathways deeply implicated in ASD, and thereby explores potential hub regulatory molecules. *NRON* (Non-coding repressor of NFAT) is a eukaryotic lncRNA approximately 2730 bp in length and its gene is located on chromosome 9q33.3. *NRON* was first discovered as the ncRNA repressor of nuclear factor of activated T cells (NFAT) [16] and its dysregulated expression was reported in peripheral blood of people with multiple sclerosis (MS) and ischemic stroke [17,18]. *NRON* has a key role in the assembly of a large ribonucleoprotein complex called the *NRON* complex and can regulate the circadian rhythm through the protein components of this complex [19]. The lncRNA highly upregulated hepatocellular carcinoma (*HULC*) gene with a length of 434 bp is located on chromosome 6p24.3 [20]. *HULC* is involved in the pathogenesis of MS [21] and plays a role in the malignant behavior of glioblastoma [22]. Upregulation of this lncRNA in liver cells disrupts the circadian rhythm of these cells through the positive regulation of the *CLOCK* gene [23]. *LINC01138* is an intergenic lncRNA which its coding gene is located on chromosome 1q21.2 with a length of 2075 bp, and its increased expression shows a positive correlations with the clinico-pathological features of glioma [24]. Furthermore, *PRMT5*, as one of the binding partners of this lncRNA, plays a role in regulating the transcription of the circadian gene *PER1* [25]. The lncRNA *MIAT*, known as *Gomafu* in humans is located at 22q12.1 with a length of 30051 bp. *MIAT* is considered as a high-risk gene for paranoid schizophrenia (SZ), and is involved in the progression of Parkinson's disease [26,27]. *TRAF3IP2* antisense transcript (*TRAF3IP2-AS1*) located at 6q21, is one of the lncRNAs modulating addiction phenotypes, and dysregulated expression of this lncRNA has been observed in dopamine neurons in the midbrain after death of cocaine addicts [28,29]. Therefore, we expected that exploring lncRNA expression in ASD would enhance our comprehension of its neurological mechanisms and underlying causes.

## 2 Methods and materials

### 2.1 Subjects

The present study was conducted using blood samples from 30 children with ASD (19 male subjects and 11 female subjects) and 41 healthy controls children (30 male subjects and 11 female subjects) referred during 2018-2022 to Behavioral Center Imam Hosein hospital (Table 1). Patients did not receive any medication in the period of one month before sampling. Inclusion criteria for the ASD group were: diagnosis based on DSM-V criteria confirmed by a senior child psychiatrist, and age between 3 and 10 years. Exclusion criteria for all participants were: history of neurological, metabolic, or autoimmune disorders; presence of a known genetic syndrome (e.g., Fragile X, Rett syndrome), and acute infectious disease at the time of sampling. Healthy controls were recruited to match the ASD group for age and sex as closely as possible. The sample size for this pilot study was determined by the availability of patients with specific characteristics who met strict inclusion criteria during the patient recruitment period in this study. Informed consent forms were signed by all participants’ parents. This study was permitted by the ethical committee of the Shahid Beheshti University of Medical Science (IR.SBMU.MSP.REC.1403.375).

**Table 1:** Demographic and clinical characteristics of the study participants.

Characteristic	ASD Group (n = 30)	Control Group (n = 41)	P-value
**Age (years), Mean ± SD**	6 ± 1.1	6.1 ± 1.5	0.36
**Age (years), Range**	4-9	4-10	-
**Sex, Male (%)**	19 (63.3%)	30 (73.2%)	0.29

### 2.2 RNA extraction and cDNA synthesis

5 ml of peripheral blood from case and controls subjects were collected in vacuum tubes of EDTA. Total RNA was extracted using the Pars tous kit (Pars tous, Cat. No.: A101231) with the spin column-based method according to the manufacturer's instructions. After total RNA extraction, the quantity and quality of the extracted RNA were evaluated by agarose gel electrophoresis and UV spectrophotometry, respectively. The extracted RNA was treated with DNase I. cDNA was synthesized from the extracted RNA using Pars tous kit (Pars tous, Cat. No.: A101161) according to the kit instructions (5 μl of total RNA, 10 μl Buffer-Mix (2x), 2 μl Enzyme Mix and 3 μl DEPC-treated water was applied in a final volume of 20 μl PCR mixture).

### 2.3 Quantitative real‐time PCR

The expression levels of target lncRNAs were determined by quantitative real-time PCR (RT-qPCR) using the ABI StepOnePlus (Applied Biosystem, Foster City, CA, USA) using 7.5 μl of SYBR® Green master mixes (Cat. No.: 1725850), 0.5 μl cDNA, 0.5 μl of each primer. Primer sequences (presented in Table 2) were designed by Oligo7. The exact cycling conditions included initial denaturation: 95°C for 10 min; 40 cycles of 95°C for 15 s and 60°C for 1 min. Beta-2-microglobulin (B2m) was applied in all samples as a reference gene and the lncRNAs values were normalized to this gene. The average ΔCT (ΔCt = Ct target gene – Ct reference gene) for the case and control groups was calculated in the case and control samples and the relative expression (fold change) of each gene was measured by 2^−ΔΔCt^ (ΔΔCt = ΔCt cases – ΔCt controls).

**Table 2:** Primers used in RT-qPCR.

lncRNAs	Primers	Primer Size	Primer Size (bp)
***NRON***	F: CGGCAGCTCGCCCTTAAATA	20	184
	R: GAACCCCCAAACCTTCCGAT	20	
***HULC***	F: ACTCTGAAGTAAAGGCCGGA	20	85
	R: TGCCAGGAAACTTCTTGCTTG	21	
***LINC01138***	F: TATTTACGAAAGCTGAAAGCG	21	217
	R: CTGCATGGGATAGGAGAAAC	20	
***MIAT***	F: CAAAGAGCCCTCTGCACTAG	20	128
	R: ACCTTGGTTACCCCTGTGATG	21	
***TRAF3IP2-AS1***	F: TTTGGCGGCTATGCAGGATT	20	189
	R: TGTCCATGTGGTATTGGGCA	20	
***B2M***	F: CCACTGAAAAAGATGAGTATGCCT	24	126
	R: CCAATCCAAATGCGGCATCTTCA	23	

### 2.4 Statistical analyses

In this study, GraphPad Prism version 8.0.2 (GraphPad Software, Inc., San Diego, CA, USA) was used for statistical analysis. The normality of the data was checked by the Kolmogorov-Smirnov test. The RNA expression levels of *NRON*, *HULC*, *LINC01138*, *MIAT* and *TRAF3IP2-AS1* were compared between patients with ASD and healthy control children using the Mann-Whitney *U* test for non-normally distributed data and the Student's t-test for normally distributed data. In addition, we utilized the Bonferroni correction for obtaining adjusted P values (q values). Pearson's correlation coefficient and standard regression test were employed to assess the correlation of gene expression two by two, as well as the correlation between the expression level of each gene and the age of affected subjects. Finally, receiver operating characteristic (ROC) was performed to determine the specificity and sensitivity of selected genes and investigate their function as potential biomarkers. P-value<0.05 was considered statistically significant.

## 3 Result

### 3.1 Expression assays

**Fig. 1:**
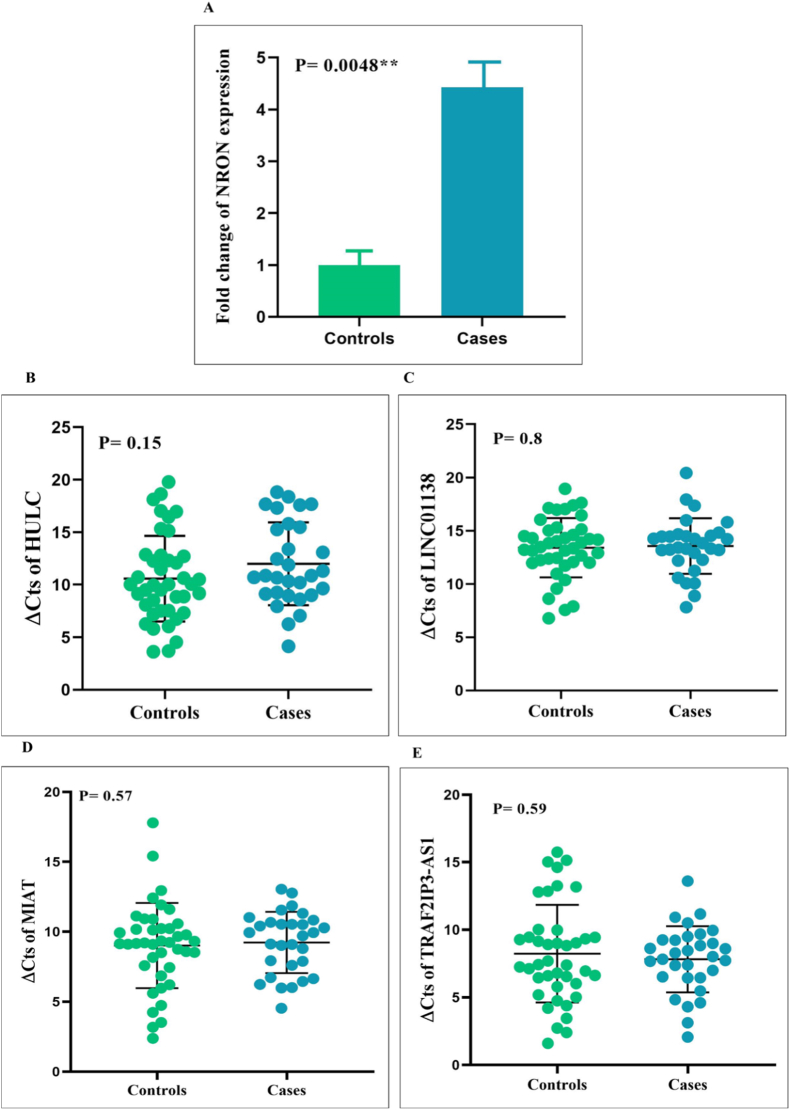
Expression analysis of lncRNAs in the patients with ASD compared to healthy children. (A) The fold change (2^−ΔΔCt^) of *NRON* expression in patients with ASD compared to HC. The ΔCt values of *HULC* (B), *LINC01138* (C), *MIAT* (D) and *TRAF3IP2-AS1* (E) and the relative expression (Fold change) of *NRON* in patients with ASD compared with healthy controls (HC) Statistical analysis was performed using Mann-Whitney *U* test (for *NRON*) and unpaired Student's t-test (for others).

The results of gene expression analyses of five lncRNAs, provided in Table 3, showed that the expression level of *NRON* was significantly increased (P = 0.004**) in patients with ASD compared to control children (Fig. 1A). Moreover, after adjustment, the P value remained significant (q-value = 0.024 for *NRON*). The expression level of *NRON* was 4.43 times higher in patients with ASD in comparison to healthy controls (Fig. 1B). However, the expression level of *HULC*, *LINC01138, MIAT, and TRAF3IP2-AS1* genes did not show any significant difference between these two groups (Fig. 1C–F). Furthermore, the expression level of each lncRNA was examined between male and female subjects and the results showed a significant increase in the expression level of *NRON* in male cases compared to male controls (P = 0.002** and q = 0.01*) but this trend was not statistically significant in female subjects (Table 3). *NRON* expression level was 6.61 times higher in boys with ASD compared to control boys (Table 3). On the other way, the expression level of lncRNAs *HULC*, *LINC01138, MIAT* and *TRAF3IP2-AS1* showed no statistically significant difference in both genders (Table 3).

**Table 3:** Relative expression of lncRNAs in total, females, and males patients with ASD and healthy controls.

Gene name	Parameters	Fold Change	ΔCt means		Up/Down	P-Value	q values
			Cases	Controls			
***NRON***	Total	4.43	10.15	12.30475	UP	0.004**	0.024*
	Female	2.37	11.32	12.57	-	0.41	0.92
	male	6.61	9.48	12.21	UP	0.002**	0.01*
***HULC***	Total	−2.66	11.99	10.58	-	0.15	0.75
	Female	−2.30	12.35	11.15	-	0.3	0.89
	male	−2.65	11.77	10.37	-	0.24	0.83
***LINC01138***	Total	−1.12	13.56	13.39	-	0.79	>0.05
	Female	−1.06	13.99	13.89	-	0.93	>0.05
	male	−1.07	13.32	13.21	-	0.89	>0.05
***MIAT***	Total	−1.16	9.23	9.01	-	0.57	>0.05
	Female	1.11	9.15	9.30	-	0.9	>0.05
	male	−1.29	9.27	8.90	-	0.52	>0.05
***TRAF3IP2-AS1***	Total	1.33	7.82	8.23	-	0.58	>0.05
	Female	1.22	8.80	9.09	-	0.83	>0.05
	male	1.58	7.24	7.91	-	0.47	>0.05

Significant values (**significant p-value <0.01, * significant q value < 0.05) are highlighted in bold. A minus (−) denotes a decrease in expression.

### 3.2 Correlation analysis

In the present study, we calculated the correlation between the expression levels of these 5 lncRNAs pairwise by Pearson's correlation test. The results of correlation analysis are included in Table 4. There was a significant positive correlation between *NRON* and *TRAF3IP2-AS1* expression levels (P = 0.0170, r = 04325). However, there was no significant correlation between the expression of other lncRNAs with each other. Moreover, examining the correlation between the age of affected children and the level of each lncRNA expression showed that there is a significant positive correlation between the expression level of lncRNAs *MIAT* (P = 0.0004***) and *TRAF3IP2-AS1* (P = 0.025*) with the age of patients with ASD but there was no noteworthy correlation between the expression of other lncRNAs and the age of patients with ASD.

**Table 4:** Pairwise correlation between expression levels of lncRNAs in cases group, correlation between expression levels of lncRNAs and age of patients.

Correlation	*HULC*		*LINC01138*		*MIAT*		*TRAF3IP2-AS1*		*NRON*	
	r	P-value	r	P-value	r	P-value	r	P-value	r	P-value
**Age**	0.16	0.39	0.15	0.40	0.60	0.0004***	0.406	0.025*	0.131	0.487
***NRON***	0.05	0.79	0.35	0.05	0.24	0.200	0.432	0.017		
***TRAF3IP2-AS1***	0.48	0.007	0.74	<0.0001	0.46	0.009**				
***MIAT***	0.17	0.34	0.25	0.17						
***LINC01138***	0.29	0.11								

### 3.3 ROC curve analysis

**Fig. 2:**
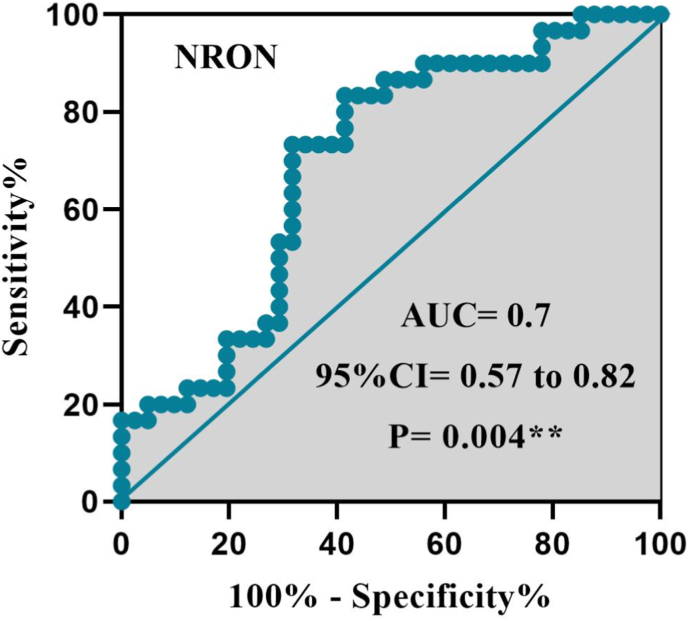
ROC curve of transcript levels of NRON in children with autism spectrum disorder. AUC: area under the curve.

We evaluated the function of the lncRNA *NRON* as a diagnostic biomarker (considering its significant difference between case and control individuals) using the ROC curve analysis. According to the obtained results, the lncRNA *NRON* gene had the area under the ROC curve (AUC) equal to 0.70 and P value = 0.004 (which is statistically significant), with a sensitivity of 73%, the specificity of 68%, and cutoff ΔCts <11.77 (Fig. 2).

## 4 Discussion

LncRNAs aberrant expression plays a key role in a variety of neurodegenerative disorders such as Alzheimer's, Huntington's, Parkinson's, spinal ataxia diseases, bipolar disorder and autism spectrum disorder [[30], [31], [32], [33]]. These transcripts may play an important regulatory role in disrupted processes in autistic people such as molecular mechanisms related to circadian rhythms and dopamine signaling pathways in the brain [7,8]. In this study, we evaluated the expression levels of lncRNAs involved in circadian rhythms (*NRON, HULC, LINC01138*) and Dopamine Signaling pathways (*MIAT* and *TRAF3IP2-AS1*) in patients with ASD and healthy control children.

Circadian rhythms are established and maintained by central and peripheral clocks. The mammalian circadian clock at a cellular level consists of at least 3 overlapping transcriptional-translational feedback loops including several clock genes and the molecular mechanisms of cell rhythm generation are independent and highly conserved in all cells [4,34,35]. Previous studies have proven that *NRON* has a key role in assembling the components of a large ribonucleoprotein complex, namely the *NRON* complex. The components of this complex include: (1) components involved in post-translational modifications and signal transduction (kinases (*CSNK1E*, *GSK3B*, *DYRK1A*), phosphatases (*PPP2R1A*) and scaffolding proteins (*IQGAP1*)), (2) components regulating protein synthesis and turnover (EIF3E, CUL4B, PSMD11 and HUWE1) and (3) components involved in nucleocytoplasmic transport (CSE1L, KPNB1 and TNPO1) [19]. The *NRON* complex components regulates the nucleocytoplasmic distribution of negative circadian clock regulators (PER/CRY) by affecting synthesis, degradation, cytoplasmic abundance and nuclear translocation of these proteins. CSNK1E, GSK3B, and DYRK1A regulate phosphorylation-dependent stability and nuclear localization of PER and CRY proteins in flies and mammals [19,36]. KPNB1 directly interacts with PER and CRY proteins and affects processes such as PER2 nuclear translocation and PER-CRY complex formation. PSMD11 acts as a determinant of nuclear CRY1:CRY2 ratio by regulating the nuclear translocation of CRY1 [19,36]. After the synthesis of PER and CRY proteins, these proteins accumulate in the cytoplasm within a few hours and enter the nucleus in complex form to suppress the binding of BMAL1-CLOCK to the promoter of its target genes [37]. Notably, BMAL1 has recently been linked to human sociability, which is often compromised in patients with ASD [38].

**Fig. 3:**
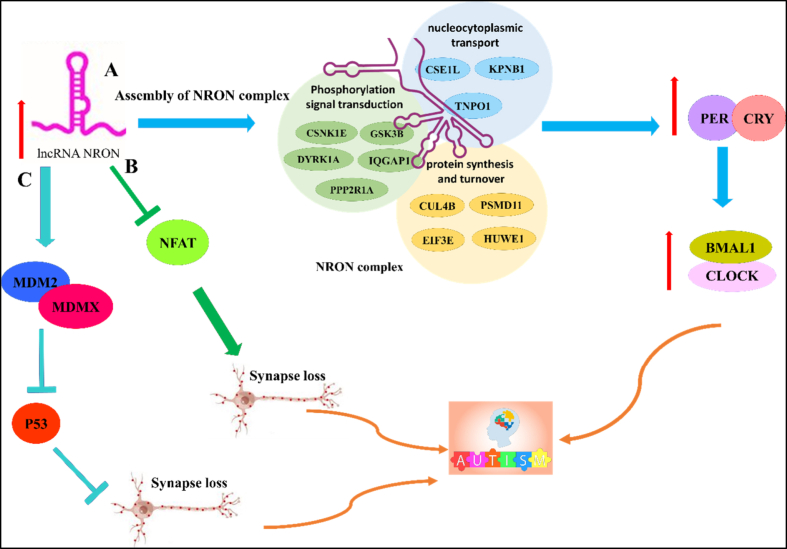
Schematic illustration of *NRON* lncRNA in ASD.

Our study showed a significant upregulation of *NRON* in ASD children compared to control children. The higher expression of *NRON* lncRNA in people with ASD may improve the assembly of *NRON* complex protein components. This increase could raise the number of *NRON* complexes in cells. As a result, it might boost the complex's role in important processes, such as post-translational modifications, the transport of negative circadian clock regulators (PER/CRY) within cells, and the transcription of key circadian clock genes (*BMAL1* and *CLOCK*). Consequently, these changes might affect the duration of the circadian period. This disruption can lead to sleep problems and other health issues in patients with ASD (Fig. 3A).

*NRON* inhibits the nuclear transfer of NFAT as a transcription factor involved in the transcription of clock proteins and genes involved in the nervous system, immune and inflammatory responses as well as neurodegeneration and prevents the transcription of its target genes [19]. Our hypothesis is that the increased expression of *NRON* in patients with ASD enhances the inhibitory effect of *NRON* on the nuclear entry of NFAT and subsequently the transcription of its target genes as autism-related genes, thus plays a role in the pathogenesis of ASD (Fig. 3B).

In addition, it has shown that the increased *NRON* expression in some studies has been associated with decreased p53 activity dependent on MDM2. MDM2 heterodimerization with MDMX, in turn, leads to increased MDM2 E3 ligase activity in p53 degradation [39]. Nowadays, the involvement of P53 in glioma and central nervous system diseases such as Alzheimer, Parkinson, cerebellar spinal ataxia and autism spectrum disorder has been identified [40]. Interestingly, p53 facilitates synaptic plasticity in the hippocampus, inhibits behaviors associated with autism, and enhances learning and memory [41], which highlights the possible role of increased *NRON* expression in the pathogenesis of ASD by reducing P53 activity through promoting MDM2: MDMX heterodimerization (Fig. 3C). This places *NRON* as a key regulator of circadian timing. Given the well-documented sleep disturbances and circadian rhythm abnormalities in ASD, our finding of *NRON* upregulation provides a plausible molecular link to this core clinical phenotype.

Notably, the significant increase in NRON was particularly evident in males, suggesting a potential sex-dependent dysregulation. Future studies with larger, sex-stratified cohorts are needed to confirm the diagnostic utility of NRON across genders.

*HULC* plays a key role in the disturbance of liver cells circadian rhythm. As a peripheral tissue, liver has a biological clock that produces daily rhythms, and *BMAL1 CLOCK* heterodimer is essential for the modulation of these rhythms in liver cells. *HULC* upregulates *CLOCK* and perturbs its rhythmical expression by binding to the 5′ untranslated region (UTR) in the *CLOCK* gene mRNA and stabilizing it in liver cells. Increased expression of *CLOCK* causes the proliferation of liver cells in in vitro and in vivo conditions and promotes liver carcinogenesis [23]. *HULC* is involved in the carcinogenesis by activation of *miR-200a-3p/ZEB1* signaling pathway [42]. In addition, increasing *HULC* expression through strengthening the PI3K/AKT/mTOR signaling pathway and increasing TNR expression by sponging *miR-128* are associated with glioma progression [22].

*LINC01138* is upregulated in glioma and increased expression of this lncRNA in glioma cells is associated with increased tumor diameter, invasion, metastasis to lymph nodes and Aerobic glycolysis (metabolism of glucose to lactate), which is a malignant adaptation in cancer and increases cell proliferation in various brain tumor microenvironments. *LINC01138* also enhances treatment resistance in glioma by upregulating SP1 expression through sponging *miR-375* [24]. PRMT5 is a member of arginine methyltransferase family that binds to the 3′ end of *LINC01138* and acts as a downstream effector of *LINC01138* in various signaling pathways. PRMT5 regulates the circadian clock in Drosophila and Arabidopsis. In mammals, PRMT5 interacts with the transcriptional repressor *CRY1* in the *PER2* promoter and suppresses the transcription of this gene by increasing the repressive activity of CRY1 and dimethylation of the *PER1* gene promoter in H4R3 [25]. The lack of significant dysregulation of HULC and *LINC01138* in our peripheral blood samples suggests that their role may be specific to glial lesions (such as glioma) or may not be detectable in the peripheral environment, emphasizing the tissue-specific nature of lncRNA expression.

Midbrain dopaminergic neurons in the substantia nigra and ventral tegmental region have a key role in controlling functions that are often compromised by ASD. The SNc DA neurons project primarily to the dorsal striatum (the caudate/putamen) thus form the nigrostriatal pathway, which regulates targeted motor behaviors and new motor skills. Disruption in this pathway may cause repetitive and stereotyped behaviors in autistic people [43]. Dopaminergic neurons in the VTA reach the PFC and the nucleus accumbens through mesocortical and mesolimbic pathways, respectively. These two pathways form the mesocorticolimbic system, which is involved in reward and motivation. Defects in mesolimbic dopaminergic signaling, such as decreased dopamine release in the PFC and decreased NAc neuronal responses, have been observed in autistic children and may lead to altered reward representation and reduced motivation to pursue rewarding events [44]. LncRNA *MIAT* regulates the expression of synaptotagmin-1 (SYT1) through sponging *miR-34-5p*, which its dysregulation has been demonstrated in PD, Alzheimer's disease, and Huntington's disease, and exerts a neuroprotective effect in a mouse model of PD [27]. Overexpression of *MIAT* in the substantia nigra and striatum of PD mice is associated with increased Parkin and tyrosine hydroxylase (TH) protein levels, increased cell viability and decreased apoptosis rate, as well as decreased motor coordination and autonomic activity [26]. *TRAF3IP2-AS1* is able to modulate the consumption phenotype in midbrain dopamine neurons (mDN), which are primary responders to drugs of abuse. Differential expression of *TRAF3IP2-AS1* has been observed in cocaine abusers and its transcript has a strong nuclear localization in dopamine cells. Also, this lncRNAy play a role in the disruption of NF–K signaling observed in cocaine abuse [28,29].

While the dopamine-related lncRNAs *MIAT* and *TRAF3IP2-AS1* were not differentially expressed in our study, their known functions in dopaminergic neurons raise speculation about a potential interaction with the circadian system. The mesolimbic dopamine pathway indicates strong circadian oscillations, and clock genes such as *BMAL1* can directly regulate dopamine synthesis [45]. Although our study does not demonstrate direct expression changes, it is possible that disruption of the circadian axis through NRON dysregulation could secondarily affect dopamine signaling and contribute to the motivational and social deficits in ASD. This *NRON*-dopamine circuit may also be relevant to other neurodevelopmental and psychiatric disorders, such as schizophrenia and bipolar disorder [46], in which both circadian and dopaminergic disruptions are prominent features.

The significant correlation between MIAT/TRAF3IP2-AS1 expression and age in the patients with ASD suggests that the regulatory landscape of these lncRNAs may evolve during development. In other words, the increasing expression of these lncRNAs with age may reflect ongoing neurodevelopmental changes in children with ASD. Unlike typically developing children, in whom certain regulatory transcripts may stabilize earlier, ASD children might exhibit protracted or altered lncRNA expression trajectories. This could mirror the delayed or aberrant maturation of dopaminergic circuits observed in ASD. Alternatively, particularly, regarding the TRAF3IP2-AS1 lncRNA, the age-related upregulation might represent a compensatory response to chronic dopaminergic dysregulation. As children with ASD grow older, their brains may attempt to normalize dopamine signaling by increasing TRAF3IP2-AS1 expression, which can be localized to dopamine neuron nuclei and modulate NF-κB signaling.

Given that ASD symptoms can change in severity and presentation with age, the correlation raises the possibility that TRAF3IP2-AS1 tracks with disease progression or accumulating neuroinflammatory burden. However, the observed correlation between expression of these lncRNAs and age of patients might be due to the confounding effect of unmeasured variables, such as medication history or behavioral interventions that could influence gene expression. In fact, without functional studies in age-stratified ASD models or longitudinal sampling, these remain speculative. A dedicated experiment measuring TRAF3IP2-AS1 expression in dopaminergic neurons derived from ASD patient iPSCs at different maturation stages would help clarify whether this age correlation is causative or incidental. Finally, the observed correlations between expressions of two lncRNAs and age, emphasizes the importance of considering age as a critical covariate in future ASD biomarker studies.

This study has limitations that should be considered. First, the sample size, although sufficient to detect large effect sizes, as observed in NRON, was limited by limited access to eligible patients with well-defined characteristics. This may affect the generalizability of our findings and limit the statistical power to detect smaller effect sizes, potentially leading to false negative results for other lncRNAs studied. Therefore, validation in larger, independent cohorts is warranted. Moreover, with small sample sizes, especially for female subgroups, the study was underpowered to detect small-to-moderate effect sizes. Thus, the negative findings (the lack of significant differences for HULC, *LINC01138*, MIAT, and TRAF3IP2-AS1) should be interpreted as inconclusive rather than truly negative. Second, although we matched groups for age and sex, we cannot completely rule out the effect of other unmeasured confounding variables, such as detailed medication histories, ASD-specific comorbidities, and symptom severity scores that were not available for all participants. Regarding sex subgroup analysis, the lack of statistical significance for *NRON* in females may well reflect Type II error rather than a true sex difference. The apparent sex-specific effect (significant only in males) should be considered hypothesis-generating only, not definitive. Third, the expression of lncRNAs was measured in peripheral blood, and although this is a clinically accessible source, it is still unclear whether these expression levels accurately reflect their expression and functional roles in the central nervous system. Finally, our study is observational and cross-sectional in nature. This study identifies an association but does not prove causation. The precise functional mechanisms through which NRON dysregulation contributes to the pathophysiology of ASD have yet to be elucidated through dedicated in vitro and in vivo functional studies.

In conclusion, our study identifies the circadian rhythm lncRNA *NRON* as a significantly dysregulated molecule in the peripheral blood of patients with ASD. Whether this peripheral expression change reflects, correlates with, or is independent of CNS *NRON* biology is unknown. Its function as a key coordinator of circadian clock components and its potential to influence neurodevelopmental pathways via NFAT and p53 make it a highly valuable central molecule and an attractive candidate for future biomarker studies. Future research should prioritize validation of *NRON* in larger, independent cohorts and use functional models to definitively determine its mechanistic contribution to ASD pathogenesis. Moreover, its expression should be assessed in patients with other pathological conditions requiring a differential diagnosis with respect to ASD.

## Ethics approval

All methods were in agreement with the ethical guidelines of the national research committee and with the 1964 Helsinki declaration and its subsequent modifications. Informed consent forms were acquired from all participants. The study protocol was verified by the ethical committee of Shahid Beheshti University of Medical Sciences.

## Role of the funding

No funding was received.

## Consent of publication

Not applicable.

## Authors’ contributions

ZSF, SGF, and AZ analyzed the data, wrote the manuscript and revised it. AZ performed the experiment and bioinformatics analysis. All the authors contributed equally and are fully aware of submission. The author(s) read and approved the final manuscript.

## Declaration of competing interest

Authors declare no conflict of interests.

## Data Availability

No data was used for the research described in the article.
