# Supplementary material for: Circadian rhythm lncRNA NRON is dysregulated in autism spectrum disorder: an observational study
Source: Biochem Biophys Rep. 2026 Jul 2;47:102698. doi: 10.1016/j.bbrep.2026.102698 (PMC13352037; doi:10.1016/j.bbrep.2026.102698)
Supplement: Multimedia component 1 [file mmc1.docx]

STROBE Statement—checklist of items that should be included in reports of observational studies

|  | Item No. | Recommendation | Page  No. | Relevant text from manuscript |
| --- | --- | --- | --- | --- |
| **Title and abstract** | 1 | (*a*) Indicate the study’s design with a commonly used term in the title or the abstract | Pages 1 and 2 | observational (case-control) study |
|  |  | (*b*) Provide in the abstract an informative and balanced summary of what was done and what was found | Page 2 | This observational (case-control) study aimed to investigate and compare the expression levels of three lncRNAs related to circadian rhythm, NRON, HULC, and LINC01138, and two lncRNAs linked to dopamine signaling, MIAT and TRAF3IP2-AS1, in the peripheral blood of 30 patients with ASD and 41 healthy controls using Real-time PCR. |
| Introduction | | | |  |
| Background/rationale | 2 | Explain the scientific background and rationale for the investigation being reported | Pages 3-4 |  |
| Objectives | 3 | State specific objectives, including any prespecified hypotheses | Page 4 | Therefore, we expected that exploring lncRNA expression in ASD would enhance our comprehension of its neurological mechanisms and underlying causes. |
| Methods | | | |  |
| Study design | 4 | Present key elements of study design early in the paper | Pages 5-6 |  |
| Setting | 5 | Describe the setting, locations, and relevant dates, including periods of recruitment, exposure, follow-up, and data collection | Page 5 | The present study was conducted using blood samples from 30 children with ASD (19 male subjects and 11 female subjects) and 41 healthy controls children (30 male subjects and 11 female subjects) referred during 2018-2022 to Behavioral Center Imam Hosein hospital. |
| Participants | 6 | (*a*) *Cohort study*—Give the eligibility criteria, and the sources and methods of selection of participants. Describe methods of follow-up  *Case-control study*—Give the eligibility criteria, and the sources and methods of case ascertainment and control selection. Give the rationale for the choice of cases and controls  *Cross-sectional study*—Give the eligibility criteria, and the sources and methods of selection of participants | Page 5 | Patients did not receive any medication in the period of one month before sampling. Inclusion criteria for the ASD group were: diagnosis based on DSM-V criteria confirmed by a senior child psychiatrist, and age between 3-10 years. Exclusion criteria for all participants were: history of neurological, metabolic, or autoimmune disorders; presence of a known genetic syndrome (e.g., Fragile X, Rett syndrome), and acute infectious disease at the time of sampling. Healthy controls were recruited to match the ASD group for age and sex as closely as possible. |
|  |  | (*b*) *Cohort study*—For matched studies, give matching criteria and number of exposed and unexposed  *Case-control study*—For matched studies, give matching criteria and the number of controls per case | Page 5 | Healthy controls were recruited to match the ASD group for age and sex as closely as possible. |
| Variables | 7 | Clearly define all outcomes, exposures, predictors, potential confounders, and effect modifiers. Give diagnostic criteria, if applicable | Page 6 | The expression levels of target lncRNAs were determined by quantitative real-time PCR (RT-qPCR) using the ABI StepOnePlus (Applied Biosystem, Foster City, CA, USA) |
| Data sources/ measurement | 8* | For each variable of interest, give sources of data and details of methods of assessment (measurement). Describe comparability of assessment methods if there is more than one group | Page 6 | The expression levels of target lncRNAs were determined by quantitative real-time PCR (RT-qPCR) using the ABI StepOnePlus (Applied Biosystem, Foster City, CA, USA) |
| Bias | 9 | Describe any efforts to address potential sources of bias | Page 7 | The RNA expression levels of NRON, HULC, LINC01138, MIAT and TRAF3IP2-AS1 were compared between patients with ASD and healthy control children using the Mann-Whitney U test for non-normally distributed data and the Student's t-test for normally distributed data. |
| Study size | 10 | Explain how the study size was arrived at | Page 5 | The sample size for this pilot study was determined by the availability of patients with specific characteristics who met strict inclusion criteria during the patient recruitment period in this study. |

Continued on next page

| Quantitative variables | 11 | Explain how quantitative variables were handled in the analyses. If applicable, describe which groupings were chosen and why | Page 7 | The normality of the data was checked by the Kolmogorov-Smirnov test. The RNA expression levels of NRON, HULC, LINC01138, MIAT and TRAF3IP2-AS1 were compared between patients with ASD and healthy control children using the Mann-Whitney U test for non-normally distributed data and the Student's t-test for normally distributed data. Pearson's correlation coefficient and standard regression test were employed to assess the correlation of gene expression two by two, as well as the correlation between the expression level of each gene and the age of affected subjects. Finally, receiver operating characteristic (ROC) was performed to determine the specificity and sensitivity of selected genes and investigate their function as potential biomarkers. P-value<0.05 was considered statistically significant. |
| --- | --- | --- | --- | --- |
| Statistical methods | 12 | (*a*) Describe all statistical methods, including those used to control for confounding | Page 7 | Whole part of statistical methods |
|  |  | (*b*) Describe any methods used to examine subgroups and interactions |  |  |
|  |  | (*c*) Explain how missing data were addressed |  |  |
|  |  | (*d*) *Cohort study*—If applicable, explain how loss to follow-up was addressed  *Case-control study*—If applicable, explain how matching of cases and controls was addressed  *Cross-sectional study*—If applicable, describe analytical methods taking account of sampling strategy |  |  |
|  |  | (*e*) Describe any sensitivity analyses |  |  |
| Results | | | | |
| Participants | 13* | (a) Report numbers of individuals at each stage of study—eg numbers potentially eligible, examined for eligibility, confirmed eligible, included in the study, completing follow-up, and analysed | Pages 7-8 | Table 3 |
|  |  | (b) Give reasons for non-participation at each stage | Not applicable |  |
|  |  | (c) Consider use of a flow diagram | Not applicable |  |
| Descriptive data | 14* | (a) Give characteristics of study participants (eg demographic, clinical, social) and information on exposures and potential confounders | Not applicable |  |
|  |  | (b) Indicate number of participants with missing data for each variable of interest | No missing data |  |
|  |  | (c) *Cohort study*—Summarise follow-up time (eg, average and total amount) |  |  |
| Outcome data | 15* | *Cohort study*—Report numbers of outcome events or summary measures over time |  |  |
|  |  | *Case-control study—*Report numbers in each exposure category, or summary measures of exposure | Page 8 | Table 3 |
|  |  | *Cross-sectional study—*Report numbers of outcome events or summary measures |  |  |
| Main results | 16 | (*a*) Give unadjusted estimates and, if applicable, confounder-adjusted estimates and their precision (eg, 95% confidence interval). Make clear which confounders were adjusted for and why they were included | Not applicable |  |
|  |  | (*b*) Report category boundaries when continuous variables were categorized | Not applicable |  |
|  |  | (*c*) If relevant, consider translating estimates of relative risk into absolute risk for a meaningful time period |  |  |

Continued on next page

| Other analyses | 17 | Report other analyses done—eg analyses of subgroups and interactions, and sensitivity analyses | Pages 10-11 | Table 4, Figure 2 |
| --- | --- | --- | --- | --- |
| Discussion | | | | |
| Key results | 18 | Summarise key results with reference to study objectives | Page 12 | Our study showed a significant upregulation of NRON in ASD children compared to control children. |
| Limitations | 19 | Discuss limitations of the study, taking into account sources of potential bias or imprecision. Discuss both direction and magnitude of any potential bias | Page 16 | This study has limitations that should be considered. |
| Interpretation | 20 | Give a cautious overall interpretation of results considering objectives, limitations, multiplicity of analyses, results from similar studies, and other relevant evidence | Page 16 | First, the sample size, although sufficient to detect large effect sizes, as observed in NRON, was limited by limited access to eligible patients with well-defined characteristics. This may affect the generalizability of our findings and limit the statistical power to detect smaller effect sizes, potentially leading to false negative results for other lncRNAs studied. |
| Generalisability | 21 | Discuss the generalisability (external validity) of the study results | Page 16 | In conclusion, our study identifies the circadian rhythm lncRNA NRON as a significantly dysregulated molecule in the peripheral blood of patients with ASD. Its function as a key coordinator of circadian clock components and its potential to influence neurodevelopmental pathways via NFAT and p53 make it a highly valuable central molecule and an attractive candidate biomarker. Future research should prioritize validation of NRON in larger, independent cohorts and use functional models to definitively determine its mechanistic contribution to ASD pathogenesis. |
| Other information | |  | | |
| Funding | 22 | Give the source of funding and the role of the funders for the present study and, if applicable, for the original study on which the present article is based | Page 17 | Role of the Funding  No funding was received. |

*Give information separately for cases and controls in case-control studies and, if applicable, for exposed and unexposed groups in cohort and cross-sectional studies.

**Note:** An Explanation and Elaboration article discusses each checklist item and gives methodological background and published examples of transparent reporting. The STROBE checklist is best used in conjunction with this article (freely available on the Web sites of PLoS Medicine at http://www.plosmedicine.org/, Annals of Internal Medicine at http://www.annals.org/, and Epidemiology at http://www.epidem.com/). Information on the STROBE Initiative is available at www.strobe-statement.org.
